# High CA125 levels as a red flag for ATTR cardiac amyloidosis

**DOI:** 10.1093/eschf/xvag199

**Published:** 2026-07-22

**Authors:** Belén Peiró-Aventín, Enrique Santas, María Ruiz-Cueto, Nerea Mora-Ayestarán, Ana Briceño, Clara Badia, Daniel de Castro, Rafael de la Espriella, Basilio Angulo-Lara, Ana Espinoza, Jorge De La Fuente García, Mikel Maeztu Rada, Ignasi Segarra Vidal, Fernando Domínguez, Esther González-López, Antoni Bayés-Genís, Pablo Garcia-Pavia, Julio Núñez

**Affiliations:** Department of Cardiology, Hospital Universitario Puerta de Hierro Majadahonda, IDIPHISA, 28222 Majadahonda, Spain; Centro Nacional de Investigaciones Cardiovasculares (CNIC), 28029 Madrid, Spain; Department of Cardiology, Hospital Clínico Universitario de Valencia, INCLIVA, 46010 Valencia, Spain; Universitat de Valencia, 46010 Valencia, Spain; Department of Cardiology, Hospital Universitari Germans Trias i Pujol, 08916 Badalona, Spain; Departamento de Medicina, Universidad Autónoma de Barcelona, 08193 Barcelona, Spain; CIBER Cardiovascular, 28029 Madrid, Spain; Department of Cardiology, Hospital Universitario Puerta de Hierro Majadahonda, IDIPHISA, 28222 Majadahonda, Spain; Centro Nacional de Investigaciones Cardiovasculares (CNIC), 28029 Madrid, Spain; Department of Cardiology, Hospital Universitario Puerta de Hierro Majadahonda, IDIPHISA, 28222 Majadahonda, Spain; Department of Cardiology, Hospital Universitari Germans Trias i Pujol, 08916 Badalona, Spain; Departamento de Medicina, Universidad Autónoma de Barcelona, 08193 Barcelona, Spain; Department of Cardiology, Hospital Universitario Puerta de Hierro Majadahonda, IDIPHISA, 28222 Majadahonda, Spain; Centro Nacional de Investigaciones Cardiovasculares (CNIC), 28029 Madrid, Spain; Department of Cardiology, Hospital Clínico Universitario de Valencia, INCLIVA, 46010 Valencia, Spain; Department of Cardiology, Hospital Universitario Puerta de Hierro Majadahonda, IDIPHISA, 28222 Majadahonda, Spain; Department of Cardiology, Hospital Universitario Puerta de Hierro Majadahonda, IDIPHISA, 28222 Majadahonda, Spain; Department of Cardiology, Hospital Universitario Puerta de Hierro Majadahonda, IDIPHISA, 28222 Majadahonda, Spain; Department of Cardiology, Hospital Universitario de Basurto, 48013 Bilbao, Spain; Department of Cardiology, Hospital General de Castellón, 12004 Castellón, Spain; Department of Cardiology, Hospital Universitario Puerta de Hierro Majadahonda, IDIPHISA, 28222 Majadahonda, Spain; Centro Nacional de Investigaciones Cardiovasculares (CNIC), 28029 Madrid, Spain; CIBER Cardiovascular, 28029 Madrid, Spain; Department of Cardiology, Hospital Universitario Puerta de Hierro Majadahonda, IDIPHISA, 28222 Majadahonda, Spain; CIBER Cardiovascular, 28029 Madrid, Spain; Department of Cardiology, Hospital Universitari Germans Trias i Pujol, 08916 Badalona, Spain; Departamento de Medicina, Universidad Autónoma de Barcelona, 08193 Barcelona, Spain; CIBER Cardiovascular, 28029 Madrid, Spain; ICREC Research Program, Instituto de Investigación Germans Trias i Pujol (IGTP), Badalona, 08916 Barcelona, Spain; Department of Cardiology, Hospital Universitario Puerta de Hierro Majadahonda, IDIPHISA, 28222 Majadahonda, Spain; Centro Nacional de Investigaciones Cardiovasculares (CNIC), 28029 Madrid, Spain; CIBER Cardiovascular, 28029 Madrid, Spain; Department of Cardiology, Hospital Clínico Universitario de Valencia, INCLIVA, 46010 Valencia, Spain; Universitat de Valencia, 46010 Valencia, Spain; CIBER Cardiovascular, 28029 Madrid, Spain

**Keywords:** CA125, Transthyretin cardiac amyloidosis, Heart failure

## Abstract

**Introduction:**

Recognition of transthyretin cardiac amyloidosis (ATTR-CA) remains challenging. In heart failure, plasmatic carbohydrate antigen 125 (CA125) is an established biomarker, reflecting inflammation and volume overload, common issues in ATTR-CA. Our aim was to evaluate the potential utility of CA125 as a ‘rule-in’ biomarker for suspected ATTR-CA.

**Methods and Results:**

This is a retrospective, multicentre, case–control study that included 626 consecutive ambulatory patients (median age 79 years, 74.8% male) from three tertiary centres. All individuals underwent 99mTc-DPD scintigraphy for suspected cardiac amyloidosis and had a CA125 determination available at the time of the evaluation. Following diagnostic workup, 451 (72%) patients were diagnosed with ATTR-CA, while 175 (28%) were ruled out for the disease. Patients with ATTR-CA showed a non-significant trend towards higher CA125 levels (16.2 [10.2–29.7] vs 14 [8–26.3] U/mL, *P* = .065). A CA125 cut-off of 40 U/mL yielded a positive predictive value of 81.7% (95% confidence interval: 78.6%–84.7%). In a multivariate logistic regression model, CA125 was an independent predictor for the diagnosis of ATTR-CA (odds ratio 2.109; 95% confidence interval: 1.197–3.717; *P* = .01). Combining CA125 (>40 U/mL) and N-terminal pro B-type natriuretic peptide (NT-proBNP) (>2500 pg/mL) significantly improved the diagnostic accuracy of the model. Patients with both biomarkers above their respective cut-offs had a three-fold higher likelihood of ATTR-CA (odds ratio: 3.38; 1.704–6.719; *P* < .001).

**Conclusions:**

In ambulatory patients with suspected ATTR-CA, a CA125 > 40 U/mL, particularly when combined with elevated NT-proBNP, supports the clinical suspicion of the disease. High CA125 levels may be useful in ATTR-CA recognition.

## Graphical Abstract

**Graphical Abstract:**
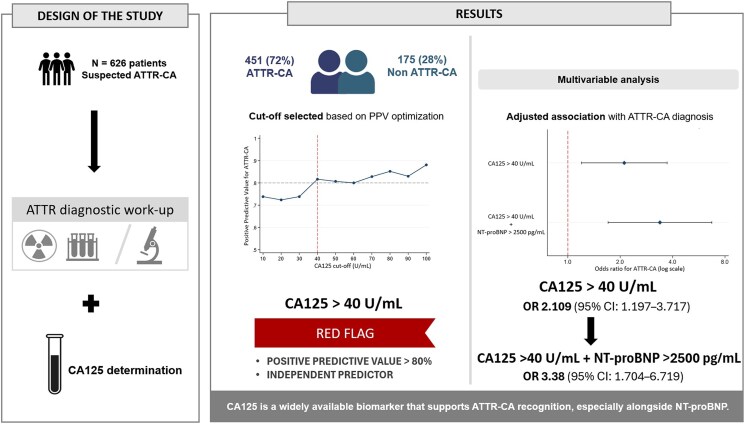
This multicentre case–control study evaluated the diagnostic value of carbohydrate antigen 125 (CA125) in ambulatory patients with suspected transthyretin cardiac amyloidosis. CA125 >40 U/mL was an independent predictor of ATTR-CA, and its combination with N-terminal pro B-type natriuretic peptide (NT-proBNP) >2500 pg/mL significantly improved diagnostic accuracy.

## Introduction

Early diagnosis of transthyretin cardiac amyloidosis (ATTR-CA), an increasingly recognized cause of heart failure (HF), remains challenging.^1,2^ Recently developed treatments, including TTR stabilizers and gene silencers, emphasize the need for timely diagnosis.^3^ Nonetheless, patients frequently experience substantial diagnostic delays, highlighting the need for reliable clinical markers assisting in early diagnosis.^1,2,4^ Carbohydrate antigen 125 (CA125), traditionally used in ovarian cancer monitoring, is now a recognized biomarker in HF, reflecting inflammatory and congestive states.^5–8^ Given that ATTR-CA patients commonly exhibit volume overload and inflammation,^9–11^ CA125 elevation may assist in identifying this condition, though its specific diagnostic utility remains uncertain. We aimed to assess CA125 as a potential biomarker to facilitate ATTR-CA recognition.

## Methods

This is a retrospective, multicentre, case–control study that included consecutive ambulatory patients who underwent 99mTc-DPD scintigraphy for suspected ATTR-CA and had a CA125 measurement available at the time of evaluation. Patients were recruited from three tertiary care centres in Spain: Hospital Universitario Puerta de Hierro Majadahonda (*n* = 548), Hospital Universitari Germans Trias i Pujol (*n* = 42), and Hospital Clínico Universitario de Valencia (*n* = 36). Hospitalized patients and those undergoing scintigraphy for indications other than suspected cardiac amyloidosis were excluded.

A definitive diagnosis of ATTR-CA was established either invasively or non-invasively, in accordance with the European Society of Cardiology recommendations.^1^

A set of demographic data, clinical characteristics, comorbidities, laboratory results, and echocardiographic parameters was collected at the time of evaluation.

The study was conducted in accordance with the principles outlined in the Declaration of Helsinki and was approved by the local institutional ethics committees of the participating centres.

### Statistical analysis

Categorical variables were compared using the chi-square test, while continuous variables were analysed using the Wilcoxon rank-sum test due to non-normal distribution. Differences in CA125 levels between patients with and without ATTR-CA were assessed accordingly. Subsequently, we aimed to determine the optimal CA125 cut-off value to achieve a positive predictive value (PPV) >80%. Once this threshold was established, its predictive performance was evaluated using multivariable logistic regression analysis, adjusting for potential confounding variables detected in the initial analysis, including age at the time of scintigraphy, sex, hypertension, diabetes mellitus, furosemide dose, estimated glomerular filtration rate, and left ventricular ejection fraction (LVEF). To enhance the PPV for the detection of ATTR-CA, we explored a combined biomarker approach incorporating CA125 and N-terminal pro B-type natriuretic peptide (NT-proBNP), an established biomarker recognized as a ‘red flag’ in ATTR-CA.^1,2^ An NT-proBNP threshold PPV > 80% was identified, and both cut-offs were applied together to stratify patients into two groups: those with both biomarkers above the thresholds and those with either one or both biomarkers below. This composite variable was then included in the logistic regression model, together with the previously described covariates.

Additionally, we assessed the incremental value of CA125 by calculating the integrated discrimination improvement (IDI) after adding it to a baseline NT-proBNP model, both unadjusted and adjusted for prespecified covariates.

A two-sided *P*-value of <.05 was considered statistically significant for all analyses. All survival analyses were performed using STATA 15.1 (StataCorp. 2015, College Station, TX: StataCorp LP).

## Results

A total of 626 patients were included, of whom 451 (72%) had a confirmed diagnosis of ATTR-CA, while 175 (28%) were ruled out for amyloidosis. Cardiac uptake grades on scintigraphy were distributed as follows: grade 0 in 175 patients (27.9%), grade 1 in 4 patients (.6%)—all with ATTR-CA confirmed by histology—grade 2 in 27 patients (4.3%), and grade 3 in 420 patients (67.2%). The median (p25% to p75%) age of the cohort was 79 years (73.9–84.4), and 468 (74.8%) were male. Baseline characteristics are summarized in *Table 1*. Compared with controls, patients with ATTR-CA were more frequently men with lower rates of cardiovascular risk factors and ischaemic heart disease. They showed larger left ventricular volumes and wall thicknesses, lower LVEF, and tricuspid annular plane systolic excursion. Additionally, ATTR-CA patients had significantly higher NT-proBNP levels.

**Table 1:** Baseline characteristics of the cohort

	Overall cohort (*N* = 626)	ATTR-CA (*N* = 451)	Controls (*N* = 175)	*P*
Age (years)	79 (73.95–84.42)	78.21 (73.47–83.61)	81.33 (74.45–86.93)	.**001**
Male (%)	468 (74.76%)	368 (81.59%)	100 (57.14%)	**<**.**001**
Hypertension (%)	442 (70.61%)	296 (65.63%)	146 (83.43%)	**<**.**001**
Diabetes mellitus (%)	195 (31.15%)	116 (25.72%)	79 (45.14%)	**<**.**001**
Coronary artery disease (%)	102 (16.29%)	65 (14.41%)	37 (21.14%)	.**041**
History of atrial fibrillation (%)	384 (61.34%)	274 (60.75%)	110 (62.86%)	.65
NYHA class (%)				.117
NYHA I	154 (24.6%)	119 (26.39%)	35 (20%)	
NYHA II	342 (54.63%)	239 (52.99%)	103 (58.86%)	
NYHA III	130 (20.77%)	93 (20.62%)	37 (21.14%)	
Dose of furosemide (mg)	40 (20–80)	40 (20–80)	40 (40–80)	**<**.**001**
CA125 (U/mL)	15.9 (9.6–29)	16.2 (10.2–29.7)	14 (8–26.3)	.065
CA125 > 40 U/mL	109 (17.41%)	89 (19.73%)	20 (11.43%)	.**014**
NT-proBNP (pg/mL)	2029 (812–4140)	2417 (983–4317)	1481 (547–3255)	**<**.**001**
NT-proBNP > 2500 pg/mL	271 (43.29%)	217 (48.12%)	54 (30.86%)	**<**.**001**
eGFR (mL/min/1.73 m^2^)	58 (41–75)	60 (45–77)	48 (31–69)	**<**.**001**
LVEDV (mL)	79 (62–106)	83 (64–107)	73 (53–104)	.**046**
LVEF (%)	56 (47.5–61.2)	55 (46.6–60)	59 (51–65)	**<**.**001**
IVS thickness (mm)	16 (14–19)	18 (15–20)	13 (12–15)	**<**.**001**
TAPSE (mm)	19 (16–21)	18 (15–21)	20 (18–22)	**<**.**001**

ATTR-CA, transthyretin cardiac amyloidosis; CA125, carbohydrate antigen 125; eGFR, estimated glomerular filtration rate; IVS, interventricular septum; LVEDV, left ventricular end-diastolic volume; LVEF, left ventricular ejection fraction; NT-proBNP, N-terminal pro B-type natriuretic peptide;NYHA, New York Heart Association; TAPSE, tricuspid annular plane systolic excursion. Statistically significant p values are shown in bold.

**Figure 1:**
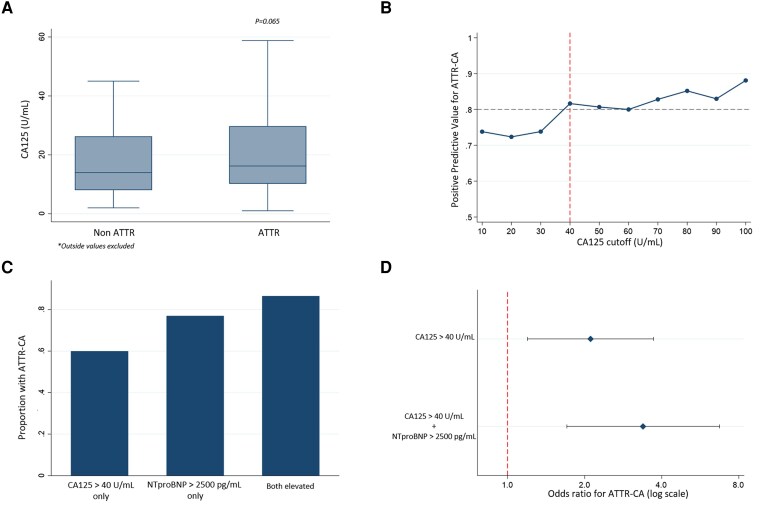
Diagnostic performance of CA125 for the recognition of ATTR cardiac amyloidosis. (A) Distribution of CA125 levels according to ATTR-CA diagnosis. (B) Positive predictive value of CA125 for ATTR-CA across different cut-off values (C) Proportion of patients with ATTR-CA according to CA125 and NT-proBNP cut-offs. (D) Forest plot representing the odds ratios and 95% confidence intervals for ATTR-CA diagnosis according to CA125 and NT-proBNP cut-offs in the multivariate models (adjusted for age, sex, hypertension, diabetes, furosemide dose, eGFR, and LVEF). ATTR-CA, transthyretin cardiac amyloidosis; CA125, carbohydrate antigen 125; eGFR, estimated glomerular filtration rate; LVEF, left ventricular ejection fraction; NT-proBNP, N-terminal pro B-type natriuretic peptide

Median CA125 was 15.9 (9.6–29) U/mL. Patients with ATTR-CA showed a trend to slightly higher CA125 (16.2 [10.2–29.7] vs 14 [8–26.3], *P* = .065). The ATTR-CA group exhibited greater dispersion, particularly noticeable in the upper range (*Figure 1A*). We then established a CA125 cut-off value of 40 U/mL, which yielded a PPV of 81.7% (95% confidence interval [CI]: 78.6%–84.7%) (*Figure 1B*). The sensitivity, specificity, and negative predictive value were 19.7% (95% CI: 16.6%–22.9%), 88.6% (95% CI: 86.1%–91.1%), and 30% (95% CI: 26.4%–33.6%), respectively. A total of 109 patients (17.4%) had CA125 levels above this threshold, including 89 patients (19.7%) with ATTR-CA and 20 patients (11.4%) without ATTR-CA (*P* = .014). Carbohydrate antigen 125 was then included as a dichotomized variable in a multivariable logistic regression model (Model 1). In this analysis, CA125 > 40 U/mL was an independent predictor of ATTR-CA, with an odds ratio (OR) of 2.109 (95% CI: 1.197–3.717; *P* = .01) (*Table 2*).

**Table 2:** Predictive factors for ATTR-CA diagnosis in Model 1 (including CA125) and Model 2 (including CA125 and NT-proBNP)

Variables	Odds ratio (95% CI)	*P*
Model 1		
CA125 > 40 U/mL	2.109 (1.197–3.717)	.**010**
Age (per 1 year increase)	1.015 (.993–1.037)	.191
Sex (female)	.512 (.338–.777)	.**002**
Hypertension	.468 (.285–.768)	.**003**
Diabetes	.589 (.393–.884)	.**010**
Furosemide dose (per 1 mg increase)	.995 (.99–.999)	.**040**
eGFR (per 1 mL/min/1.73 m^2^ increase)	1.014 (1.004–1.025)	.**007**
LVEF (per 1% increase)	.969 (.952–.986)	**<**.**001**
Model 2		
CA125 > 40 U/mL and NT-proBNP > 2500 pg/mL	3.38 (1.704–6.719)	**<**.**001**
Age (per 1 year increase)	1.015 (.993–1.037)	.191
Sex (female)	.503 (.331–.766)	.**001**
Hypertension	.458 (.279–.754)	.**002**
Diabetes	.602 (.399–.906)	.**015**
Furosemide dose (per 1 mg increase)	.994 (.989–.999)	.**022**
eGFR (per 1 mL/min/1.73 m^2^ increase)	1.014 (1.004–1.025)	.**007**
LVEF (per 1% increase)	.969 (.953–.987)	.**001**

ATTR-CA, transthyretin cardiac amyloidosis; CA125, carbohydrate antigen 125; eGFR, estimated glomerular filtration rate; LVEF, left ventricular ejection fraction. Statistically significant p values are shown in bold.

In a sensitivity analysis, to further enhance the diagnostic performance for ATTR-CA, we incorporated NT-proBNP alongside CA125 in the multivariable model (Model 2). In our cohort, an NT-proBNP cut-off of 2500 pg/mL resulted in a PPV of 80.1% (95% CI: 76.9%–83.2%), and a total of 271 patients (43.3%) had NT-proBNP levels exceeding this threshold. By combining both biomarkers, patients were classified into two groups: those with CA125 and NT-proBNP levels above their respective cut-offs and all others. Eighty-nine patients (14.2%) had elevated levels of both biomarkers. When added to the previous model, this new combined variable was a highly significant and independent predictor for ATTR-CA diagnosis, yielding an OR of 3.38 (95% CI: 1.704–6.719; *P* < .001) (*Table 2*, *Figure 1C* and *D*).

Furthermore, the inclusion of CA125 significantly improved model discrimination compared with the NT-proBNP-only model, yielding an IDI of .009 (SE .0033; *P* = .008), indicating a .9% absolute improvement in the separation of predicted probabilities between patients with and without the outcome. These findings were consistent after adjustment for covariates (IDI .009, SE .0035; *P* = .008).

## Discussion

To our knowledge, this is the first study to explore the usefulness of CA125 in patients with suspected ATTR-CA. Although median CA125 levels were numerically higher in patients with ATTR-CA, this difference did not reach statistical significance. However, we found that a CA125 threshold of >40 U/mL was associated with a two-fold higher likelihood of an ATTR-CA diagnosis. The PPV of CA125 was enhanced when combined with NT-proBNP. The combination of elevated CA125 and NT-proBNP levels in patients with suspected ATTR-CA might be useful for recognizing patients with ATTR-CA.

Diagnosing ATTR-CA remains challenging due to non-specific symptoms and a complex diagnostic pathway, often involving multiple imaging modalities and invasive procedures.^1,2^ Early recognition is crucial to halt HF progression and maximize therapeutic benefits.^3,12^ Yet, delays remain prevalent, with nearly 60% of patients misdiagnosed initially. Many patients consult multiple physicians and undergo extensive evaluations before reaching the diagnosis.^4^

Biomarkers play a central role in cardiovascular diagnosis, notably in HF. However, in ATTR-CA, biomarkers such as troponins and NT-proBNP have primarily shown prognostic and monitoring value rather than strong diagnostic capabilities.^13^ While elevated NT-proBNP or persistently raised troponins have been proposed as red flags of ATTR-CA, recent studies indicate their utility predominantly lies in excluding rather than confirming the disease.^14^ For example, data from Mayo Clinic patients undergoing 99mTc-PYP scintigraphy showed limited PPV (≤70%) for both markers.^15^

Carbohydrate antigen 125 is a widely accessible biomarker, robustly validated in HF due to its strong correlation with congestion, especially interstitial fluid accumulation/third space.^6–8^ It complements NT-proBNP and potentially surpasses it in conditions characterized by predominant volume overload, such as right-sided HF or severe tricuspid regurgitation.^16,17^ Additionally, CA125 elevation is linked to systemic inflammation markers (e.g. TNFα, IL-1β, IL-6), suggesting it participates actively in inflammatory pathways relevant to HF pathophysiology.^6,9,10^

These mechanisms support the hypothesis that CA125 could be a useful biomarker in ATTR-CA, although evidence to date has been lacking. Indeed, inflammation is significant in ATTR-CA, with myocardial inflammation detectable on biopsy in approximately one-third of patients.^11^ Additionally, restrictive haemodynamics with elevated intracardiac pressures and systemic congestion further support CA125’s potential relevance.^18,19^ Recently, elevated CA125 levels were independently associated with higher mortality in light chain (AL) amyloidosis patients, strengthening its potential utility in ATTR-CA.^20^

The relatively low sensitivity of CA125 > 40 U/mL observed in our cohort indicates that it cannot be used as a standalone screening biomarker for ATTR-CA. Instead, its potential clinical value lies in its high PPV, supporting a possible role in the ‘rule-in’ process and in raising clinical suspicion of ATTR-CA, particularly when interpreted alongside NT-proBNP.

The study has several limitations that should be acknowledged. First, its retrospective design and the potential selection bias of a cohort referred for scintigraphy due to suspected ATTR-CA. Second, the CA125 threshold was derived from the study cohort and therefore requires external validation in independent populations. Finally, the study focused on the diagnostic performance of CA125 and did not assess longitudinal outcomes or its potential prognostic value in ATTR-CA.

In conclusion, CA125, especially levels above 40 U/mL in conjunction with elevated NT-proBNP, is a potential novel red flag for suspecting ATTR-CA and may enhance early detection of ATTR-CA.

## Declarations

### Data Availability

No data were generated or analysed for this manuscript.

### Pre-registered Clinical Trial Number

None supplied.
